# Magnetic Resonance Imaging Quantification of the Liver Iron Burden and Volume Changes Following Treatment With Thalidomide in Patients With Transfusion-Dependent *ß*-Thalassemia

**DOI:** 10.3389/fphar.2022.810668

**Published:** 2022-02-18

**Authors:** Jinlian Che, Tianying Luo, Lan Huang, Qiyang Lu, Da Yan, Yinying Meng, Jinlan Xie, Weihua Chen, Jiangming Chen, Liling Long

**Affiliations:** ^1^ Department of Radiology, First Affiliated Hospital of Guangxi Medical University, Nanning, China; ^2^ Department of Radiology, Seven Affiliated Hospital of Guangxi Medical University (Wuzhou Gongren Hospital), Wuzhou, China; ^3^ Department of Hematology, Seven Affiliated Hospital of Guangxi Medical University (Wuzhou Gongren Hospital), Wuzhou, China; ^4^ NHC Key Laboratory of Thalassemia Medicine (Gaungxi Medical University), Nanning, China

**Keywords:** magnetic resonance imaging, thalidomide, thalassemia, liver iron concentration, liver volume measurement

## Abstract

Clinical trials have indicated that thalidomide could be used to treat thalassemia, but evidence of changes in liver iron burden and liver volume during thalidomide treatment is lacking. This study aimed to evaluate the liver iron burden and volume changes following thalidomide treatment in patients with transfusion-dependent *ß*-thalassemia. A total of 66 participants with transfusion-dependent *ß*-thalassemia were included in this prospective cohort study between January 2017 and December 2020. Patients were treated with thalidomide (150–200 mg/day) plus conventional therapy. Liver volume, liver R2*, and hepatic muscle signal ratio (SIR)_T1 and SIR_T2 were measured with magnetic resonance imaging (MRI), and serum ferritin, hemoglobin, erythrocyte and platelet counts, and liver function were measured at baseline and at the 3rd and 12th months. Adverse events were also noted. Patients showed progressive increase in hemoglobin, erythrocyte, platelet count, SIR_T1, and SIR_T2 during the 12-months follow up. Serum ferritin, R2*, and liver volume progressively decreased during the follow up. The R2* value had a significantly positive correlation with serum ferritin, and SIR_T1 and SIR_T2 had a significantly negative correlation with serum ferritin. No serious adverse events were observed. This study showed that thalidomide could potentially be used to successfully treat patients with transfusion-dependent *ß*-thalassemia; the liver iron burden and liver volume could be relieved during treatment, and the MRI-measured R2*, SIR_T1, and SIR_T2 may be used to noninvasively monitor liver iron concentration.

## 1 Introduction

Thalassemia is one of the most common genetic disorders worldwide, and it is likely to remain a serious global health concern for the foreseeable future. *ß*-thalassemia is one of the most common forms of severe thalassemia in many parts of Asia (Weatherall, 2018). *ß*-thalassemia, which is caused by more than 200 point mutations, results in a decrease in the production of *ß*-globin chains, leading to abnormal erythropoiesis and varying degrees of anemia (Rund and Rachmilewitz, 2005). In recent years, the most critical change in clinical diagnosis is a new simplified classification that helps guide clinical management of thalassemia intermedia into non-transfusion-dependent thalassemia and thalassemia major into transfusion-dependent thalassemia based on their requirement of regular blood transfusions for survival (Viprakasit and Ekwattanakit, 2018). Blood transfusion therapy can stop the progression of the disease by providing normal red blood cells, inhibiting ineffective red blood cell production, and reducing extramedullary hematopoiesis. However, repeated transfusions can cause iron accumulation and overload (Shah et al., 2019). In addition, hepatomegaly and splenomegaly are more common in hemoglobin E *ß*-thalassemia and are associated with lower pre-transfusion hemoglobin levels (Mettananda et al., 2019), Papadaki et al. (2003) reported that the 70.5% of hepatomegaly and 48.6% of splenomegaly were found by abdominal ultrasonography in patients with thalassemia (92 patients) and sickle-cell anemia (13 patients). Iron overload causes most of the mortality and morbidity associated with thalassemia, and iron deposition occurs in visceral organs, such as the liver, heart, and endocrine glands (pituitary and pancreas), causing tissue damage and ultimately organ dysfunction and failure (Rund and Rachmilewitz, 2005).

As an anti-inflammatory, anti-angiogenic, and immunomodulatory drug (Sherbet, 2015), thalidomide has been reported to induce fetal hemoglobin (HbF) synthesis and proliferation of erythroid cells (Aerbajinai et al., 2007; Moutouh-de Parseval et al., 2008). Li et al. (2018) reported that thalidomide had a significant effect in patients with thalassemia, whether transfusion-independent or transfusion-dependent. Furthermore, a recent study (Chen et al., 2017) of nine patients with thalassemia showed that treatment with thalidomide significantly improved the hemoglobin level and reduced spleen size, and another study (Chen et al., 2020) suggested that thalidomide could potentially be useful for the management of thrombocytopenia in patients with hypersplenism. A multicenter study (Yang et al., 2020) reported that thalidomide had significant therapeutic effects in patients with *ß*-thalassemia with a sustained response.

However, few studies have examined changes in iron stores *in vivo* during thalidomide treatment, as the liver is the primary organ for iron deposition, and the liver iron concentration is the most important indicator of iron content in the body; therefore, it is important to evaluate the liver iron burden and volume changes in patients with transfusion-dependent *ß*-thalassemia, which can help comprehensively assess the iron deposition state in the body. The potential impact of thalidomide treatment on liver volume and liver iron deposition can be evaluated to provide a theoretical basis for the selection of subsequent treatment decisions and clinical management for patients with transfusion-dependent *ß*-thalassemia.

At present, liver biopsy is the gold standard for evaluating liver iron overload, but liver biopsy is not an ideal method for detecting liver iron deposition because of the possibility of sampling error and its invasive nature, and it is not suitable for repeated sampling. Serum ferritin is most commonly measured as an indicator of iron stores; ferritin levels below 2,500 mg/ml are associated with lack of cardiac dysfunction and improved survival (Hoffbrand et al., 2003). However, the serum ferritin levels are highly unreliable, particularly when inflammation or liver disease is present, and cannot accurately reflect the state of iron deposition in the body (Brittenham et al., 1993). Magnetic resonance imaging (MRI) provides a possible technique for direct noninvasive measurement of hepatic iron stores, and it has been reported that liver iron concentration can be measured either with relaxometry methods T2*/T2 or signal intensity ratio techniques (Fernandes, 2018). John et al. (Wood et al., 2005) illustrated that both R2 and R2* can accurately measure hepatic iron concentration throughout the clinically relevant range of hepatic iron concentration using appropriate MRI acquisition techniques. Amany et al. (Mousa et al., 2016) suggested that pituitary to fat and liver to muscle signal intensities are useful, noninvasive tools for the early diagnosis of pituitary and liver iron overload.

Therefore, to the evaluate liver iron burden, the R2* value of the liver and the ratio of liver-to-muscle signal intensities were measured with MRI before and during thalidomide treatment, simultaneously, the liver volumes were accurately measured with MRI, and the correlations of serum ferritin and R2* and the ratio of liver-to-muscle signal intensities were analyzed. Accordingly, we evaluated the potential effects of thalidomide therapy on liver volume and liver iron deposition.

## 2 Methods

### 2.1 Study Design and Participants

The ethics committee of our institution (Gongren Hospital, Wuzhou City, Guangxi, China) approved the protocol for this study (2017–043), which adhered to the principles of the Declaration of Helsinki, and informed written consent was obtained from all participants. This study was a part of phase-II clinical trial evaluating safety and efficacy of thalidomide in patients with transfusion-dependent *ß*-thalassemia (Chinese Clinical Trial Registry, registration number: ChiCTR1800015702), MRI follow-up was performed at baseline and during treatment in our department.

**FIGURE 1:**
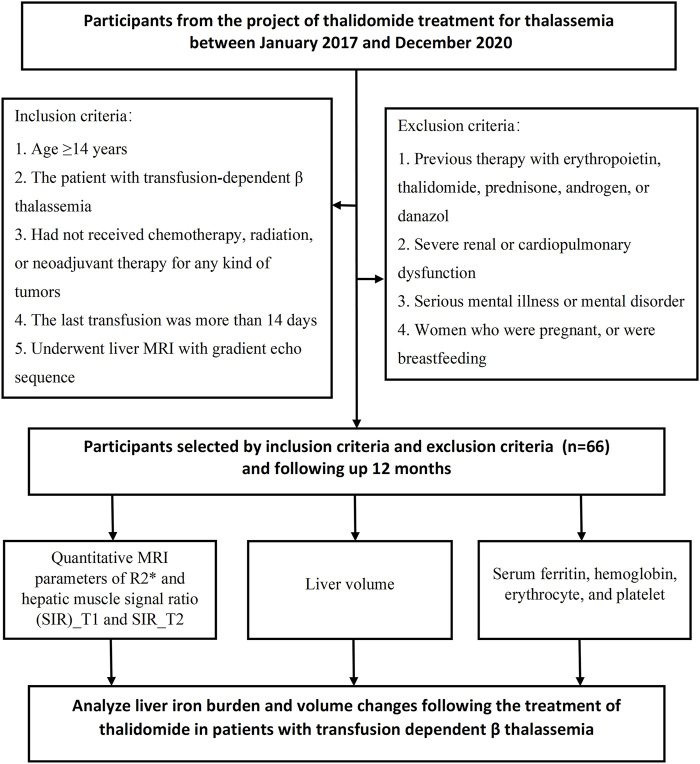
Flowchart of the prospective study cohort. A total of 66 participants were included in this study.

This prospective cohort study included consecutive patients with transfusion-dependent *ß*-thalassemia at a single regional large-scale comprehensive medical center (Gongren Hospital, Wuzhou City, Guangxi, China) between January 2017 and December 2020 (Figure 1). The inclusion criteria were: 1) age ≥14 years; 2) a diagnosis of transfusion-dependent *ß*-thalassemia made using accepted clinical and genetic methods, but unable to afford regular iron chelation (patients should stop iron chelation treatment 2 weeks before enrollment); 3) no provision of chemotherapy, radiation, or neoadjuvant therapy for any type of tumor; 4) eastern cooperative oncology group (ECOG) physical score between 0 and three points; 5) last transfusion having taken place >14 days earlier; 6) liver MRI with a gradient echo sequence. The exclusion criteria were: 1) previous therapy with erythropoietin, thalidomide, prednisone, androgen, or danazol; severe renal dysfunction; 2) serious mental illness or mental disorder; 3) severe cardiopulmonary dysfunction; 4) women who were pregnant or intended to become pregnant in the near future or who were breastfeeding; and 5) participation in another clinical trial.

The diagnostic criteria for thalassemia were as follows: microcytic hypochromic anemia, erythrocyte target cells observed in peripheral blood smears, red blood cells with reduced osmotic fragility, elevated HbF level, and slightly elevated HbA2 level (b-thalassemia). Genetic analysis confirmed the presence of a mutant gene associated with *ß*-thalassemia. DNA was extracted from patients’ peripheral blood leukocytes and mutations of the *ß*-globin gene were analyzed by polymerase chain reaction (PCR)-reverse dot blot. Seven single nucleotide polymorphisms (SNPs) of HBG2 (rs7482144), HBS1L-MYB (rs9399137 and rs4895441), and BCL11A (rs4671393, rs10189857, rs1427407 and rs11886868) were tested by Sanger sequencing of PCR products.

### 2.2 Interventions and Thalidomide Treatment

Participants with *ß*-thalassemia were treated with blood transfusions but were unable to afford regular iron chelation because of economic or other reasons. Patients with a hemoglobin level <60 g/L were administered red blood cell transfusion; when they had a platelet count <20×10^9^/L or hemorrhage, they received platelet transfusion. All participants received thalidomide instead of conventional medical treatment, but during thalidomide treatment, if patients with continuous high serum ferritin or low hemoglobin (Hb < 60 g/L), they will be treated with iron chelation or blood transfusion. Thalidomide was administered before sleep at an initial dose of 50–75 mg/day. If no adverse reactions were observed, the dose was increased the following day to the target dose of 150–200 mg/d, and thalidomide was administered continuously for 12 months. The dose of thalidomide was decided with reference to the consensus of Chinese experts on the diagnosis and treatment of primary myelofibrosis (2015 version) (Xue et al., 2015). These patients did not receive iron chelation treatment during thalidomide treatment.

### 2.3 Follow-Up and Outcome Measures

All patients were followed up for 12 months. The following investigations were carried out at 3, 6, and 12 months after the start of therapy: serum ferritin, hemoglobin level, erythrocyte count, platelet count, bilirubin level, alanine aminotransferase (ALT), and aspartate aminotransferase (AST). The requirement for red blood cell transfusion or platelet transfusion during the follow-up period was also analyzed as an outcome measure. The occurrence and severity of any adverse events (such as thrombosis, neurotoxicity, rash, somnolence, constipation) were evaluated. For routine blood tests (XN-2000 Hematology Autoanalyzer, Sysmex, Kobe, Japan), venous blood samples were obtained from the median cubital vein in the morning, awake, and fasting state.

### 2.4 Liver Iron Burden and Volume Changes

#### 2.4.1 MRI Examination

Liver MRI was performed using a 3.0-T MRI scanner (Discovery, General Electric Medical Systems, Milwaukee, WI) with a 4-element torso coil, before thalidomide treatment (baseline) and at 3 and 12 months of treatment. The scan ranged from the top of the diaphragm to the lower margin of the liver. The gradient echo sequence was used to measure liver R2*, echo time (TE) = 0.94, 1.85, 2.71, 3.62, 4.48, 5.39, 6.25, 7.16, 8.02, 8.93, 9.79, and 10.07 ms; repetition time (TR) = 200 ms; flip angle = 20°; field of view (FOV) = 36 cm × 38 cm; matrix = 160 × 160; bandwidth = 83 kHz; thickness = 6 mm; T1-weighted imaging (T1WI) was performed with TR = 3.7 ms, TE = 2.0 ms, flip angle = 12°, section thickness = 6 mm, layer spacing = 2.0 mm, FOV = 36 cm × 38 cm; coronal half Fourier single excitation fast spin echo sequence T2-weighted imaging (T2WI) with TR = 1800.0 ms, TE = 90.0 ms, flip angle = 180°, section thickness = 6 mm, layer spacing = 2.0 mm, FOV = 36 cm × 38 cm; and fat suppression T2WI sequence with TR = 6,315.0 ms, TE = 78.0 ms, flip angle = 111°, section thickness = 6 mm, layer spacing = 2.0 mm, FOV = 36 cm × 38 cm.

#### 2.4.2 MRI Image Analysis

The original Digital Imaging and Communications in Medicine files for liver MRI were transferred to CMRTools (Cardiovascular Imaging Solutions, London, United Kingdom) for post-processing of R2* and image analysis. Regions of interest (ROIs) were drawn in the left and right lobes of the liver with areas of no less than 3 cm^2^, excluding obvious vessels and biliary ducts; the left and right lobes of the liver were measured three times with ROIs of similar size, and the average amounts were calculated. Two physicians (radiologists with 5 years of experience in abdominal MR imaging) who were blinded to the clinical data independently measured the MRI images.

The gradient echo (R2*) and spin-echo (R2) images were fitted to monoexponential equations with a variable offset (Wood et al., 2005): S (TE) = Ae^−TE . R2*^ + C (Eq. 1). C is a constant that is necessary to compensate for contributions from instrumentation noise and effects from iron-poor species such as blood and bile. The same ROI as R2* was copied to the liver’s T1WI and T2WI for measuring the signal strength of T1 and T2, and concurrently, the signal strength of the same slice vertical ridge muscle was measured, and the hepatic muscle signal ratio (SIR)_T1 and SIR_T2 were calculated.

ADW4.5 (General Electric Medical Systems) was used to measure the liver volume, and the 3D-LAVA-FLEX sequence images were input into the 3D volume measurement protocol of the post-processing workstation. From the top of the liver to the lower edge of the liver, the outline of the liver was manually drawn layer by layer on the transverse image to avoid hepatic fissure, ligamentum teres hepatis, gallbladder, left and right branches of the portal vein, main portal vein, and inferior vena cava. Finally, the volume calculation protocol automatically calculated the liver volume.

### 2.5 Statistical Analysis

SPSS 22.0 (IBM, Armonk, NY, United States) was used for statistical analysis. The Kolmogorov–Smirnov method was used to test whether the data exhibited a normal distribution. Normally distributed continuous variables are expressed as mean ± standard deviation, and non-normally distributed continuous variables are expressed as median (range); for the variances at baseline, 3^rd^ month, and 12th month, one-way analysis of variance was used to compare the normally distributed data, and Bonferroni correction was performed for multiple comparisons. Friedman’s rank test or the Wilcoxon test was used to assess differences in non-normally distributed data. The correlations of serum ferritin with R2*, SIR_T1, SIR_T2, and liver volume were calculated using the Spearman correlation coefficient. Interobserver agreement for MRI measurements of liver volume, SIR_T1, SIR_T2, and R2* between the two radiologists were analyzed by calculating the intraclass correlation coefficient (ICC). The following guidelines were used for interpretation of the ICC inter-rater agreement measures: less than 0.40, poor; between 0.40 and 0.59, fair; between 0.60 and 0.74, good; between 0.75 and 1.00, excellent. Statistical significance was set at *p* < 0.05.

## 3 Results

### 3.1 Demographic and Clinical Characteristics of the Patients

A total of 66 patients with transfusion-dependent *ß*-thalassemia were included in the study with ages of 18.89 ± 6.37 years (range, 14.0–40.0 years); 42 (63.6%) were male and 24 (36.4%) female. For genotyping, 24.2% (16/66) had a β0/β0, 6.1% (4/66) had a β0/β+, 60.6% (40/66) had a β0/β++, 4.5% (3/66) had a β+/β++, 4.5% (3/66) had a β++/β++, and simultaneous *a* globin mutation showed in three patients. The demographic and clinical characteristics of the patients at baseline and during the follow-up period are shown in Table 1. The serum ferritin level was not significantly different between the baseline and 3^rd^ month [3,955.10 (1000.98–18545.34) ng/ml vs 3,619.23 (937.75–17365.33) ng/ml, *p* > 0.05]; in the 12th month, the serum ferritin level was 3,389.34 (220.22–12,312.36), which was significantly lower than that at baseline and at the 3^rd^ month (*p* < 0.05). The hemoglobin and erythrocyte levels were significantly increased from 71.73 ± 13.52 g/dl and 2.98 ± 0.53 (10^12^/L) to 107.47 ± 18.61 and 5.02 ± 0.85 (10^12^/L) during the 12 months follow-up period (*p* < 0.05); the platelet count was not significantly different (*p* > 0.05), but it showed an increasing tendency with the prolongation of thalidomide treatment, from 291.50 (62.00–1294.00) 10^9^/L at baseline to 365.00 (35.00–1006.00) 10^9^/L at the 12th month; the ALT and AST values significantly changed from 30.25 (5.00–217.70) U/L and 34.25 (11.00–176.30) U/L to 20.90 (6.70–160.80) U/L and 20.00 (11.70–135.70) U/L, respectively, during the 12-months follow-up period (*p* < 0.05). Before thalidomide treatment (baseline), the median annual red blood cell transfusion rate was 36.0 U (range 2.0–96.0 U), and during the 12-months follow up, the median annual red blood cell transfusion rate was 2.0 U (range 0–23.0 U), showing a significant decrease (Z = -0.71, *p* < 0.05); 30 of these patients did not receive blood transfusion within 12 months of thalidomide treatment (Figure 3).

**TABLE 1:** Liver volume, hepatic muscle signal ratio, R2*, and clinical characteristics of the 66 patients with transfusion dependent *ß*-thalassemia before thalidomide treatment and at the 3^rd^ month and 12th month after treatment.

	Baseline	3rd month	12th month	*p* Value
Liver volume (mm^3^)	1850.84 ± 368.66	1788.68 ± 372.22	1723.57 ± 370.55	0.217^##^
SIR_T1	0.23 ± 0.12	0.30 ± 0.15	0.36 ± 0.15	<0.001^##^
SIR_T2	0.10 ± 0.03	0.14 ± 0.07	0.16 ± 0.09	<0.001^##^
R2* (Hz)	702.21 (95.32–1789.21)	595.00 (102.33–1261.00)	464.33 (220.22–1012.00)	<0.001
Serum ferritin (ng/ml)	3,955.10 (1000.98–18,545.34)	3,619.23 (937.75–17,365.33)	3,389.34 (220.22–12,312.36)	0.023^##^
Hemoglobin [g/dL]	71.73 ± 13.52	94.89 ± 17.97	107.47 ± 18.61	<0.001^##^
Erythrocyte (10^12^/L)	2.98 ± 0.53	4.54 ± 0.95	5.02 ± 0.85	<0.001^##^
Platelet (10^9^/L)	291.50 (62.00–1294.00)	303.50 (34.00–1358.00)	365.00 (35.00–1006.00)	0.191^#^
Direct bilirubin (μmol/L)	9.16 ± 3.24	10.00 ± 3.80	9.62 ± 3.55	0.473^##^
Indirect bilirubin (μmol/L)	39.48 ± 21.48	38.35 ± 24.66	37.18 ± 26.37	0.894^##^
ALT (U/L)	30.25 (5.00–217.70)	33.50 (6.20–133.10)	20.90 (6.70–160.80)	0.003^#^
AST (U/L)	34.25 (11.00–176.30)	27.35 (12.10–133.00)	20.00 (11.70–135.70)	<0.001^#^

^##^ one-way analysis of variance (ANOVA); ^#^ Friedman’s rank test.

SIR, hepatic muscle signal ratio; ALT, alanine aminotransferase; AST, aspartate aminotransferase.

### 3.2 MRI Analysis During Follow-Up

**FIGURE 2:**
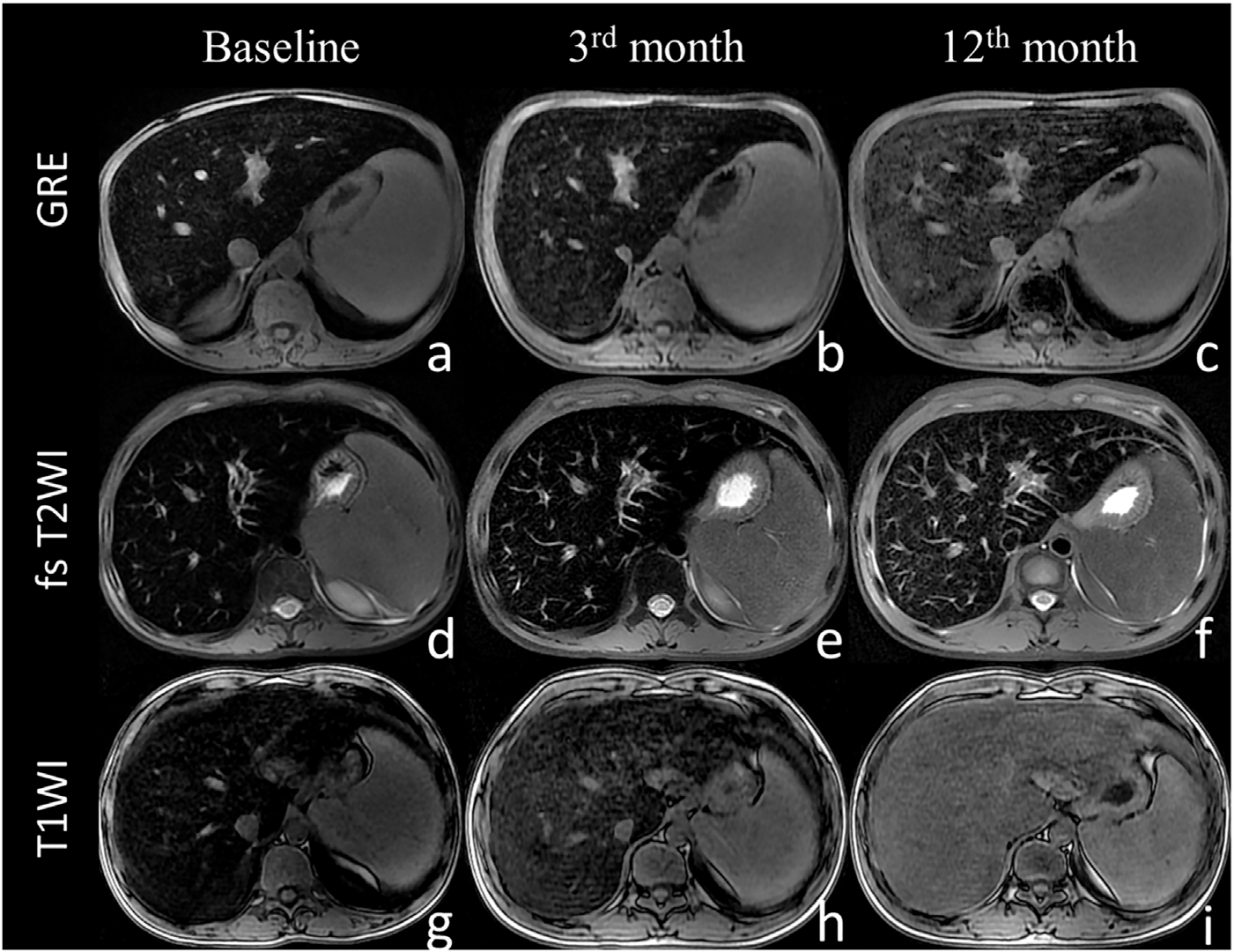
The liver magnetic resonance imaging signal intensity with the sequence of gradient echo **(A–C)**, fast spin echo sequence T2-weighted imaging **(D–F)** and T1-weighted imaging **(G–I)** at baseline **(A,D,G)**, 3 months **(B,E,H)**, and 12 months **(C,F,I)**. The liver MRI signal intensity increased with thalidomide treatment during the 12 months of follow up.

**FIGURE 3:**
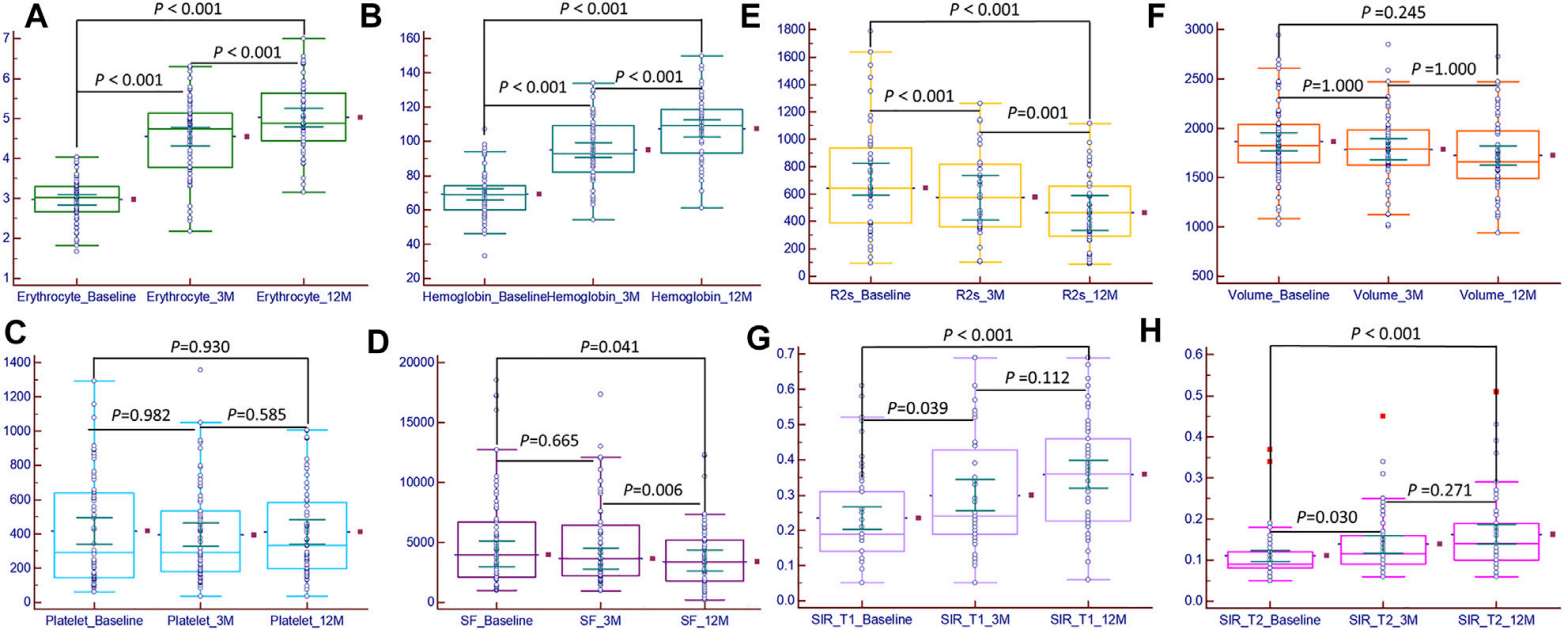
Bar chart of magnetic resonance imaging metrics and clinical characteristics at baseline, 3^rd^ month, and 12th month. The erythrocyte **(A)**, hemoglobin **(B)**, hepatic muscle signal ratio (SIR)_T1 **(G)**, and SIR_T2 **(H)** significant increased (*p* < 0.05); the platelet count **(C)** increased, but the difference was not significant (*p* > 0.05); serum ferritin **(D)** and R2* **(E)** significant decreased (*p* < 0.05); the liver volume **(F)** decreased, but the difference was not significant (*p* > 0.05).

The liver SIR_T1 and SIR_T2 during the 12-months follow up showed an upward trend in the patients treated with thalidomide and were significantly higher at 3 and 12 months than at baseline (all *p* < 0.05) (Table 2). The liver R2* was 702.21 (95.32–1789.21) Hz, 595.00 (102.33–1261.00) Hz, and 464.33 (220.22–1012.00) Hz at baseline, 3^rd^ month, and 12th month, respectively, showing a significant decrease (*p* < 0.05). The liver volume showed a trend of improvement from baseline to 12 months, but the difference was not significant, from 1850.84 ± 368.66 mm^3^ at baseline to 1723.57 ± 370.55 mm^3^ at 12 months (*p* > 0.05, Tables 1, 2; Figures 2, 3).

**TABLE 2:** Multiple comparisons (*p* values) of the baseline, 3^rd^ month, and 12th month groups for liver volume, hepatic muscle signal ratio, R2*, and clinical characteristics.

	Baseline Vs. 3rd month	Baseline Vs. 12th month	3rd month Vs 12th month
Liver volume (^##^)	1.00	0.245	1.00
SIR_T1 (^##^)	0.039	<0.001	0.112
SIR_T2 (^##^)	0.030	<0.001	0.271
R2* (^#^)	<0.001	<0.001	0.001
Serum ferritin (^#^)	0.665	0.041	0.006
Hemoglobin (^##^)	<0.001	<0.001	<0.001
Erythrocyte (^##^)	<0.001	<0.001	<0.001
Platelet (^#^)	0.982	0.930	0.585
Direct bilirubin (^##^)	0.665	1.00	1.00
Indirect bilirubin (^##^)	1.00	1.00	1.00
ALT (^#^)	0.038	0.015	0.004
AST (^#^)	0.177	<0.001	0.001

^##^ Bonferroni correction was performed for multiple comparisons; ^#^ Wilcoxon test.

SIR, hepatic muscle signal ratio; ALT, alanine aminotransferase; AST, aspartate aminotransferase.

**FIGURE 4:**
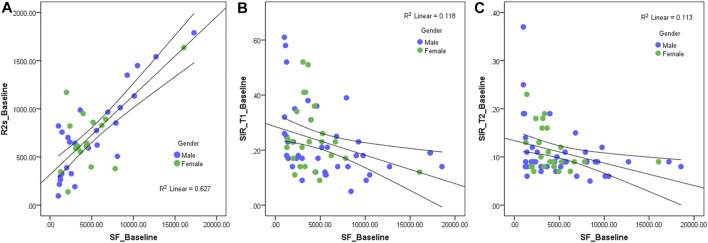
Scatter diagram of serum ferritin and R2* **(A)**, hepatic muscle signal ratio (SIR)_T1 **(B)**, and SIR_T2 **(C)** at baseline. The R2* value has a significantly positive correlation with serum ferritin (r = 0.649, *p* < 0.05), and the SIR_T1 and SIR_T2 have a significantly negative correlation with the serum ferritin (r = −0.387 and -0.336, *p* < 0.05).

At baseline, 3^rd^ month, and 12th month, there were significant positive correlations between serum ferritin and R2*, liver volume, ALT, and AST (at baseline, r = 0.649, 0.386, 0.505, and 0.425; *p* < 0.05; at the 3^rd^ month, r = 0.614, 0.442, 0.659, and 0.609; *p* < 0.05; at the 12th month, r = 0.638, 0.329, 0.498, and 0.400; *p* < 0.05); there were significant negative correlations between serum ferritin and SIR_T1 and SIR_T2 (at baseline, r = -0.387 and -0.336; *p* < 0.05; at the 3^rd^ month, r = -0.482 and -0.527; *p* < 0.05; at the 12th month, r = -0.564 and -0.468; *p* < 0.05). At baseline, there was no significant correlation between serum ferritin and erythrocyte level (r = -0.005, *p* > 0.05), but in the 3rd and 12th months, there were significant negative correlations between serum ferritin and erythrocyte level (r = -0.507 and -0.348, *p* < 0.05) (Table 3; Figure 4).

**TABLE 3:** Correlation analysis between serum ferritin and MRI metrics and clinical laboratory indicators during thalidomide treatment.

Variables	Baseline		3rd month		12th month	
	r value	*p* value	r value	*p* value	r value	*p* value
R2*	0.649	<0.001	0.614	<0.001	0.638	<0.001
SIR_T1	−0.387	0.001	−0.482	0.001	−0.564	<0.001
SIR_T2	−0.336	0.007	−0.527	0.000	−0.468	0.001
Liver volume	0.386	0.002	0.442	0.003	0.329	0.018
ALT	0.505	<0.001	0.659	0.000	0.498	<0.001
AST	0.425	<0.001	0.609	0.000	0.400	0.005
Erythrocyte	−0.005	0.968	−0.507	0.000	−0.348	0.013

SIR, hepatic muscle signal ratio; ALT, alanine aminotransferase; AST, aspartate aminotransferase.

### 3.3 Consistency Analysis

**FIGURE 5:**
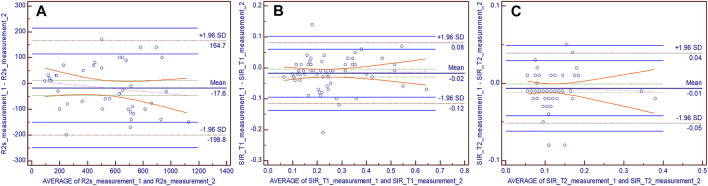
Bland–Altman difference plots for R2* **(A)**, hepatic muscle signal ratio (SIR)_T1 **(B)**, and SIR_T2 **(C)** measurements generated by two independent readers. The dotted red lines demarcate the 1.96 standard deviations (SD), and the blue lines depict their 95% prediction limits.

The ICC values of liver volume, SIR_T1, SIR_T2, and R2* were 0.953 (95% confidence interval: 0.925–0.971), 0.923 (0.877–0.952), 0.917 (0.867–0.948), and 0.946 (0.902–0.971), respectively. Bland–Altman plots showed good interobserver agreement of the SIR_T1, SIR_T2, and R2* measurements between the two observers (Figure 5).

### 3.4 Adverse Reactions

Adverse events were reported in 10 patients, all of which were mild in severity and tolerated by the patients. The most common side effects were gastrointestinal reactions, which included nausea (3/66), emesis (2/66), and ventosity (2/66), followed by a rash (2/62) and menstruation disorders (1/24); these symptoms were transient and recovered after temporary drug discontinuation or symptomatic treatment. During the 12-months follow up, none of the patients developed peripheral neurotoxicity with intermittent numbness of both lower limbs. Intracranial or visceral bleeding or thrombosis did not occur in any patient.

## 4 Discussion

In this study, the results indicated that patients with transfusion-dependent *ß*-thalassemia had progressive and significant increase in hemoglobin and erythrocyte counts; although there was no significant difference in the platelet count, it tended to increase during the 12 months of thalidomide therapy. Furthermore, in the 12th month, the serum ferritin levels were significantly lower than those at baseline and at the 3^rd^ month, and the liver SIR_T1 and SIR_T2 showed a significant upward trend, while the liver R2* significantly decreased. In addition, there were significant positive correlations between serum ferritin and R2*, liver volume, ALT, and AST, and there were significant negative correlations between serum ferritin and SIR_T1 and SIR_T2. No serious adverse events occurred during the course of the study, and thalidomide was well tolerated.

Patients with transfusion-dependent *ß*-thalassemia (thalassemia major) may have high blood transfusion requirements beginning at an early age. Current management of this disease includes the use of regular blood red cell transfusions, iron chelation therapy, and bone marrow transplantation (Lawson et al., 2003). As a consequence of the regular transfusion regimen being body iron accumulation (Cohen et al., 2008), iron overload causes most of the mortality and morbidity associated with thalassemia. Iron deposition occurs in visceral organs (mainly in the liver, heart, and endocrine glands), causing tissue damage and ultimately organ dysfunction and failure (Rund and Rachmilewitz, 2005). In order to make appropriate chelation decisions and accurate conclusions regarding patient adherence to chelation therapy (Puliyel et al., 2014), available guidelines (Guidelines for the management of transfusion-dependent thalassemia, 3rd edition) for the management of patients with *ß*-thalassemia major recommend annual examinations of liver iron overload (Musallam et al., 2013).

β-thalassemia increases the levels of γ globin substitute for absent *ß* globin and mitigate the relative *a* chain excess to ameliorate the *ß* hemoglobin disorder, hence stimulating increase in γ globin transcription and HbF production in patients with *ß*-hemoglobinopathies, which has been considered as a therapeutic strategy. Thalidomide has a mechanism of action similar to that of other immunomodulatory agents that have been used for multiple myeloma treatment and induce HbF production (Aerbajinai et al., 2007) A previous study showed that thalidomide is capable of inducing HbF in a patient with thalassemia major. Moreover, Chen et al. (2017) demonstrated that patients with transfusion-dependent thalassemia treated with thalidomide experienced an optimal curative effect, and all patients who received uninterrupted thalidomide therapy had rapid and progressive increases in hemoglobin values and no longer needed transfusion. Li et al. (2018) also reported that thalidomide had a significant effect in patients with thalassemia intermedia; among patients who require transfusion, it was terminated or decreased by > 50% after 1 month of treatment. Consistent with previous studies, our study illustrated that hemoglobin and erythrocyte count significantly increased during 12 months of thalidomide therapy, and the median annual red blood cell transfusion rate significantly decreased [from baseline at 36.0 U (range 2.0–96.0 U) to the 12th month at 2.0 U (range 0–23.0 U)]. A prospective cohort study (Chen et al., 2020) reported that the platelet count progressively increased during 12 months of thalidomide therapy, and the results of our study showed that although the platelet count did not significantly change, it tended to increase during the 12-months follow up.

To our knowledge, no study has reported on the iron burden during thalidomide treatment. Theoretically, the body iron burden would be relieved because thalidomide treatment reduces the amount of blood transfusions in patients with *ß*-thalassemia major. Serum ferritin is the most commonly used measure for the diagnosis and monitoring of total body iron overload, but it cannot accurately reflect the status of iron deposition in the organs (Aydinok, 2018). Angelucci et al. (2000) demonstrated that the liver is a major organ of total body iron stores in patients with thalassemia major and suggested that in patients with transfusion-related iron overload, repeated determinations of the hepatic iron concentration can provide a quantitative means of measuring the long-term iron balance. Iron shortens T1, T2, and T2* relaxation times measured with MRI and leads to darkened images in the presence of iron; therefore, the use of MRI relaxation time techniques to estimate liver iron concentration has been widely used in clinical practice (Brittenham and Badman, 2003; Voskaridou et al., 2004), offering great potential for widely accessible, noninvasive estimation of hepatic iron concentration. R2* is the reciprocal of T2*, which is directly proportional to iron and demonstrates the most promising results; most investigators have described a linear increase in R2* with iron (Clark et al., 2003; Ruefer et al., 2017). John et al. (Wood et al., 2005) illustrated that MRI R2 and R2* mapping could accurately estimate hepatic iron concentration in transfusion-dependent thalassemia and sickle cell disease. Gandon et al. (1994), Gandon et al. (2004) showed the ratio of liver MRI signal intensity to the signal obtained in a skeletal muscle within the same field of view, a tissue that is not influenced by iron overload, which is a noninvasive and feasible method that has been used clinically to quantify hepatic iron concentration by comparing results of quantification of iron through liver biopsies with biochemical determination. Amany et al. (Mousa et al., 2016) indicated that the liver-to-muscle signal intensities were significantly correlated with serum ferritin and bilirubin levels. Similarly, our study revealed that serum ferritin was positively correlated with liver R2* and negatively correlated with the hepatic muscle SIR_T1 and SIR_T2, further indicating that the degree of liver iron concentration is correlated with the state of iron deposition in the whole body, and liver R2*, SIR_T1, and SIR_T2 can reflect the state of iron overload in the whole body to a certain extent. Serum ferritin levels did not significantly decrease until the 12th month after thalidomide treatment, but significant reduction in the liver R2* and significant increase in SIR_T1 and SIR_T2 were found at both the 3rd and 12th months of treatment. This indicates that the whole-body iron overload was alleviated after a period of treatment, and thalidomide treatment can reduce liver iron deposition in the 3^rd^ month which predates the decrease in serum ferritin level (it alleviated until the 12th month of treatment). This also indicates that changes in liver iron deposition are inconsistent with changes in serum ferritin levels over time. In the course of thalidomide treatment, there may have been a reduction in liver iron deposition when there was no significant change in serum ferritin levels. The evaluation of MRI R2*, SIR_T1, and SIR_T2 may provide a reference for whether patients need to be treated with iron chelation during thalidomide treatment.

In *ß*-thalassemia major, to compensate for anemia, an increasing rate of extramedullary hemopoiesis would occur, leading to increased production of abnormal red blood cells and their clearance; hence, hypersplenism and increases in spleen size are also observed (Kolnagou et al., 2013). Chen et al. (2020) reported that all 31 patients (hepatic cirrhosis, n = 19 and thalassemia, n = 12) who received thalidomide treatment showed progressive decrease in spleen length during a 12-months follow up. In addition, increase in liver size could also be observed in patients with *ß*-thalassemia major; Fischer et al. (1999) revealed increase in iron concentration of 0.6–11.0 mg/g in the liver and in the size of the liver of approximately 18% per 1 mg/g liver in patients with *ß*-thalassemia major. In this study, we accurately measured the liver volume of such patients (*n* = 66) before and after thalidomide treatment using MRI and found a progressive decrease in liver volume during the 12 months of follow up, and the liver volume was positively correlated with serum ferritin levels, indicating that body iron overload may result in increased liver volume. Simultaneously, this study also found that the ALT and AST values decreased during treatment, and both the ALT and AST values were positively correlated with the serum ferritin level, indicating that during thalidomide treatment, liver function could be restored with reduction in body iron deposition.

Safety analysis revealed no severe adverse reactions associated with thalidomide treatment in this study. Although it is well known that thalidomide has a teratogenic effect and cannot be used by pregnant women, adverse reactions to thalidomide are rare in other patients (Vargesson, 2015). Indeed, we observed that few side effects were associated with gastrointestinal reactions, including nausea, emesis, and ventosity, followed by a few rash and menstruation disorders. Thus, we believe that thalidomide would be well tolerated by non-pregnant patients with *ß*-thalassemia.

This study had several limitations. First, this was an observational study, and no control group was included; our sample may have been susceptible to selection bias, and the level of evidence is inferior to that of randomized controlled trials. Second, this was a single-center study. Although this study enrolled 66 patients with transfusion-dependent *ß*-thalassemia, the generalizability of the findings needs further validation by multicenter studies. Third, the 12-months follow-up period was relatively short, and the long-term and adverse effects of thalidomide were not investigated. The success of thalidomide treatment might lie in fewer transfusions needed and increase in hemoglobin, erythrocyte, and platelet counts, which may contribute to decrease in body iron deposition, providing a theoretical basis for the use of MRI technology to explore changes in liver iron concentration during thalidomide treatment. Nevertheless, as this was a preliminary study, the conclusion should be interpreted with caution, and prospective, randomized controlled trials with large sample sizes are needed to validate our findings.

In summary, our findings suggest that thalidomide could constitute a novel pharmacologic therapy for patients with *ß*-thalassemia, reducing body iron deposition and the incidence of hepatomegaly while increasing hemoglobin, erythrocyte, and platelet counts. The MRI-measured R2*, SIR_T1, and SIR_T2 can be used to noninvasively monitor liver iron concentration, which may provide more information on whether patients with transfusion-dependent *ß*-thalassemia need to receive iron chelation treatment during treatment with thalidomide.

## Data Availability

The original contributions presented in the study are included in the article/supplementary material, further inquiries can be directed to the corresponding authors.
